# Factors influencing aversion to specific electrodiagnostic studies

**DOI:** 10.1002/brb3.240

**Published:** 2014-07-22

**Authors:** Nivedita U Jerath, Scott B Strader, Chandan G Reddy, Andrea Swenson, Jun Kimura, Edward Aul

**Affiliations:** 1 Department of Neurology, Carver College of Medicine, The University of IowaIowa City, Iowa, USA; 2 Department of Neurosurgery, Carver College of Medicine, The University of IowaIowa City, Iowa, USA; 3 Iowa City Veterans Affairs Medical Center, Department of NeurologyIowa City, Iowa, USA

**Keywords:** Aversion, electrodiagnostic studies, electromyography, muscle, nerve, nerve conduction studies, pain

## Abstract

**Objective:**

To compare the degree of discomfort caused by nerve conduction studies (NCS) versus needle electromyography (EMG), and to determine what factors predict aversion to one test or the other.

**Methods:**

Two hundred patients underwent both EMG and NCS, and were asked to indicate which test was more uncomfortable. Responses were then correlated with demographic information, testing characteristics, and medical histories to identify any notable associations.

**Results:**

Of the 200 patients, 58.5% (117) of the patients found the NCS more uncomfortable than EMG. Sixty-one percent (11/18) of the younger patients (18–29 years old) found EMG more uncomfortable (*P* = 0.08), whereas 68% (40/59) of the older patients (age greater than 60 years old) found NCS more uncomfortable (*P* = 0.05). Sixty-seven percent (14/21) of the patients whose BMI was less than 22 kg/m^2^ rated EMG as more uncomfortable (*P* = 0.01). Sixty-nine percent (27/39) of the patients whose BMI was greater than or equal to 38 found the NCS more uncomfortable (*P* = 0.02). A positive correlation existed between NCS discomfort and number of nerves tested. 67% (35/52) of the patients with polyneuropathy found NCS more uncomfortable.

**Conclusion:**

Nerve conduction studies are more uncomfortable than needle EMG in the majority of patients, and predictions regarding which test will be more uncomfortable for a given patient are possible.

## Introduction

Nerve conduction studies (NCS) and needle electromyography (EMG) are invaluable electrodiagnostic (EDX) tools in the diagnosis of neuromuscular disorders. Unfortunately, both procedures involve subjecting the patient to physical discomfort, including electric shocks and needle punctures. Despite the utility of the data gained from the testing, the prospect of undergoing such testing may be daunting to both the patient and the referring clinician, and in some cases the patient may refuse the test. However, prior studies have suggested that both pharmacologic and nonpharmacologic strategies may be useful in decreasing perceived levels of discomfort in patients undergoing EDX studies, and that some patients benefit simply by being better informed about the test prior to its performance (Buckelew et al. 1992; Richardson et al. 1994; El-Salem and Shakhatreh 2008). By our empirical observation, we have the impression that many of our patients with a neuropathy find the NCS to be more uncomfortable than the needle EMG. In the current study, we wish to test this hypothesis by a simple survey to compare discomfort of NCS to that of EMG. In addition, we try to identify factors that might predict a more painful experience for an individual patient, and thereby we can employ these strategies in an efficient manner.

Multiple studies have attempted to identify determinants of pain experienced during EDX; these have chiefly, although not exclusively, focused on the needle EMG portion of the examination (Gans and Kraft 1977; Khoshbin et al. 1987; Richardson et al. 1994; Strommen and Daube 2001). Factors that have been correlated with increased pain perception during the testing include older age, female sex, higher levels of self-assessed pain related to patients' medical conditions, higher levels of baseline anxiety based on personality testing, use of a monopolar needle, and needle insertion technique. A 2004 study by Wee et al. (2004) found that pain experienced during the needle EMG study correlated with pain experienced during the NCS; their data showed no significant relationship between sex, age, BMI, or needle size and levels of pain perception. This study also found that EMG was perceived as more painful than NCS, but other data have suggested the opposite to be true (Gans and Kraft 1977). As noted above, based on empirical observations from our laboratory, we hypothesized that a majority of patients would find the NCS to be more uncomfortable than the needle EMG, and we sought to determine whether or not that was the case. We also sought to determine whether or not previously identified predictors of testing-related pain influenced patients' aversion for one test or the other, as well as to identify new factors, not previously studied to our knowledge, that may predict whether or not a patient would find the EMG or the NCS more uncomfortable. These included the absolute number of nerves and muscles tested, the inclusion of specific nerves or muscles in the test, as well as the referral and/or confirmed diagnosis (e.g., neuropathy).

## Materials and Methods

We surveyed 200 patients who underwent testing in the EMG Laboratory at the University of Iowa Hospitals and Clinics between February and June 2011. Patients were excluded from the study if they were less than 18 years old, did not undergo both testing modalities, were encephalopathic, required an interpreter to understand English, or were currently a prison inmate. Immediately following completion of testing, the patients were given a paper survey with a single question: “During your testing today, which of the two procedures caused you the most discomfort?” The patient was then instructed to check a box indicating either the “nerve conduction studies (shock testing)” or the “electromyogram (needle testing).” Patients were required to answer the question “on the spot.” The surveys were distributed and collected either by the clinical neurophysiology fellow or one of the laboratory's nerve conduction technologists. Patients for whom data were collected gave signed informed consent, and the study methodology was approved by the University of Iowa Institutional Review Board. A retrospective chart review was then performed for all patients surveyed, collecting demographic information and medical history. Demographic information included age, sex, BMI, referral diagnosis, EMG diagnosis, and whether or not the referral diagnosis was confirmed by EDX. In addition, testing characteristics were recorded, including the presence or absence of the clinical neurophysiology fellow or resident at any point in the testing. The number and names of the nerves and muscles tested were also recorded. Motor studies and sensory studies of the same nerve were counted separately. F-wave studies were also considered to be separate and were added to the count of the total number of nerves tested for each patient. The data were then analyzed using descriptive statistics and Fisher's exact test to determine and compare the percentages of patients who had an aversion to needle EMG versus those who had an aversion to the NCS, and any associations between the aversion and specific demographic factors, test characteristics, or medical history items.

Nerve conduction studies and needle EMG were performed using 9200MEB Nihon Kohden equipment. All of NCS were performed by registered nerve conduction technologists, and for some of the studies, the clinical neurophysiology fellow participated as well. Each patient had an electrophysiologic investigation under the same laboratory conditions. All studies used 0.1-ms electrical pulses; stimuli were applied with an intensity of 20% above the current necessary to evoke a maximal SNAP or CMAP. Stimulus intensity during the NCS ranged from 20 to 100 milliamperes, with stimulus duration of 0.1–1 ms.

Needle EMG was performed by an experienced attending physician in either neurology or physiatry, or by a trainee (fellow or resident) under the supervision of an attending physician. The Teca Elite disposable concentric bipolar needle electrodes (37 mm × 26G and 50 mm × 26G sizes) were used.

## Results

Demographic characteristics of the patients studied are presented in the table. 51.5% of the participants were male. The mean age was 50.75 years, with a range of 18–85. Mean BMI of the participants was 31.3 kg/m^2^, with a range of 16.5–68.5 kg/m^2^.

The most common referral diagnosis was lumbosacral radiculopathy (27%), followed by polyneuropathy (19%), cervical radiculopathy (14%), carpal tunnel syndrome (9%), ulnar neuropathy (6%), myopathy (4%), and other diagnoses (21%). The most common EMG result was a normal study (44%), followed by polyneuropathy (25%), carpal tunnel syndrome (10%), lumbosacral radiculopathy (6%), ulnar neuropathy, cervical radiculopathy, peroneal neuropathy (all 2%), and other diagnoses (8%). Of the 200 surveyed patients, 51 (25.5%) had their referral diagnosis confirmed by the EDX.

For all patients surveyed, the mean number of nerves tested with NCS was 9.4 (range 3–18), and the mean number of muscles tested with needle EMG was 4.6 (range 1–16). The clinical neurophysiology fellow participated in 109 (54.5%) of the examinations, and a neurology resident participated in 89 (44.5%). For the NCS, the most common studies performed were ulnar motor, ulnar sensory, tibial motor, and sural sensory, each of which were included in 71.5% of examinations. For needle EMG, the most common muscle tested was the tibialis anterior, which was included in 65.5% of examinations, followed by the first dorsal interosseous of the hand and the gastrocnemius, both of which were included in 45% of examinations.

**Figure 1:**
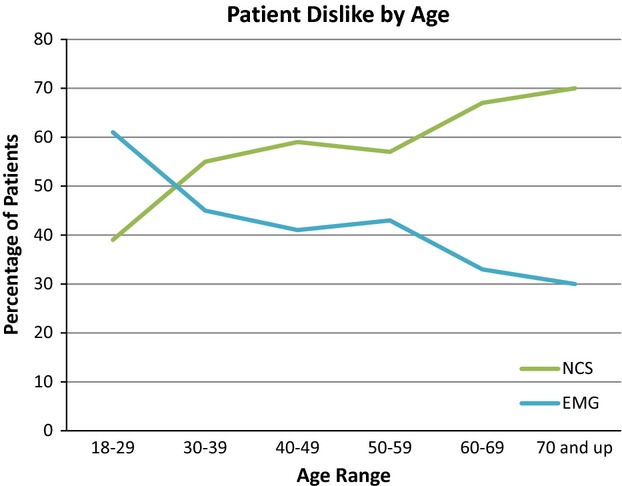
Patient aversion by age.

**Figure 2:**
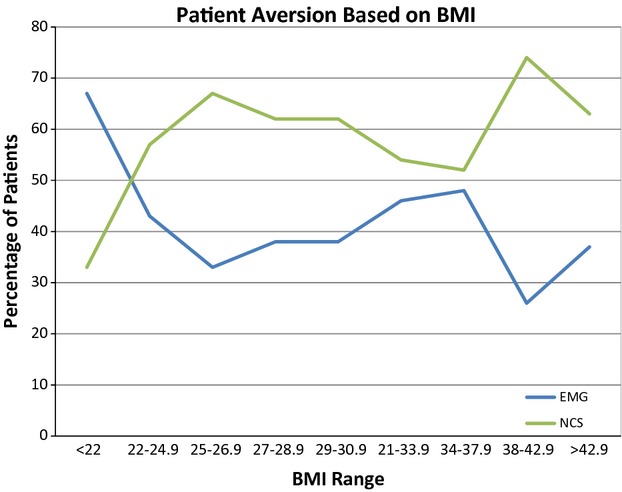
Patient aversion by BMI.

Of the 200 patients who participated in the study, 58.5% (117) found the NCS more uncomfortable, and 41.5% (83) found the needle EMG more uncomfortable. A trend was observed with respect to age: 61% (11/18) of the younger patients (18–29 years old) found EMG more uncomfortable (*P* = 0.08), whereas 68% (40/59) of the older patients (age greater than 60 years old) found NCS more uncomfortable (*P* = 0.05) (Fig. 1). No clear deviation from baseline was noted with respect to patient gender. Sixty-seven percent (14/21) of the patients whose BMI was less than 22 kg/m^2^ rated EMG as more uncomfortable (*P* = 0.01). Sixty-nine percent (27/39) of the patients whose BMI was greater than or equal to 38 found the NCS more uncomfortable (*P* = 0.02) (Fig. 2).

**Figure 3:**
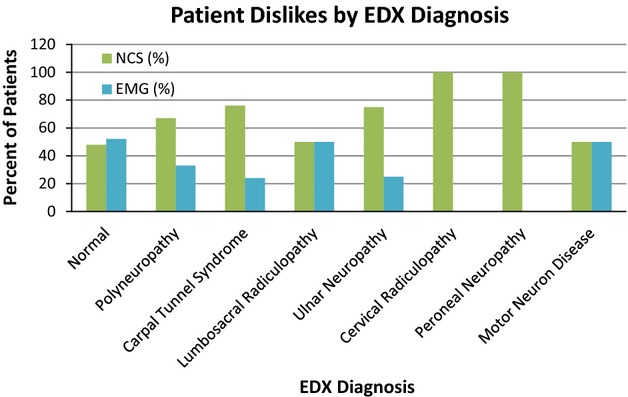
Patient aversion by EMG diagnosis.

**Figure 4:**
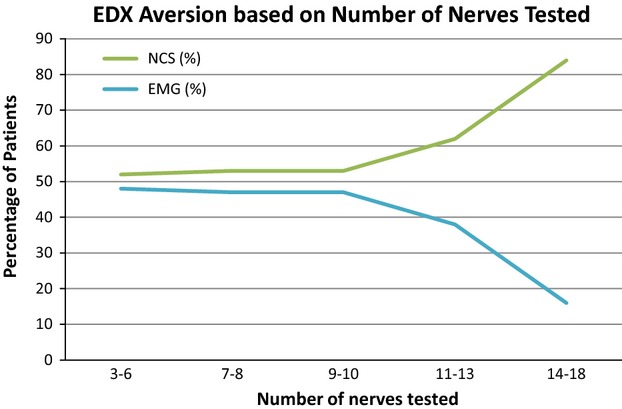
Patient aversion based on number of nerves tested with NCS.

**Figure 5:**
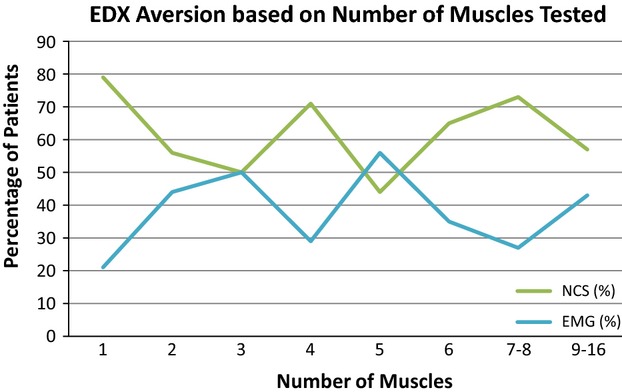
Patient aversion based on number of muscles tested with EMG.

Patients whose EDX were normal rated the EMG more uncomfortable than the NCS, and patients whose EDX suggested polyneuropathy were more likely to find the NCS more uncomfortable than the general study population (Fig. 3). Confirmation or lack of confirmation of the referral diagnosis was not associated with a deviation from baseline aversion. Patients who had 10 or fewer nerves tested with NCS found either the NCS or the EMG more uncomfortable with approximately equal frequency. However, if 11 or more nerves were tested, a positive association between discomfort of the NCS and the number of nerves tested was noted (Fig. 4). The relationship between patient aversion and the number of muscles tested with EMG was less clear (Fig. 5). Patients who had zero to three more nerves tested with NCS than muscles tested with EMG rated the EMG as more uncomfortable; in patients who had four or more nerves tested than muscles, the NCS were rated as more uncomfortable. Interestingly, patients who had more muscles tested with needle EMG than nerves with NCS clearly rated the NCS as more uncomfortable, although the number of patients in this group (9) was small.

No statistically significant difference exists between the discomfort of an EMG performed by a trainee or an attending (*P* = 0.7261): patient aversion to an attending's EMG was 44% (18/41) compared to a trainee's (fellow or resident) EMG, which was 41% (65/159).

For specific nerves, only the median-ulnar sensory comparison study was associated with greater dislike of NCS than the baseline study population. Although the data are not shown in the figure due to the small population, it should be noted that six of seven patients who underwent ulnar inching studies at the elbow found the NCS to be more uncomfortable. Patients who had the gastrocnemius or biceps brachii tested with needle EMG rated the EMG as more uncomfortable with a higher frequency than the baseline study population, although these groups still rated the NCS as more uncomfortable overall. Patients who had the iliopsoas tested rated the NCS as more uncomfortable with a higher frequency than the baseline study population.

## Discussion

To our knowledge, this is the largest study to determine the factors that influence aversion to specific EDX. Several observations are worthy of comment. A clear relationship between patient age and dislike of the NCS was noted. Younger patients (those under the age of 30) found the EMG more uncomfortable, and in patients older than 30, advancing age was associated with a greater likelihood of finding the NCS more uncomfortable. While female gender has been associated with greater levels of pain during the testing as noted above, gender does not seem to influence aversion of one modality over the other. Patients with lower BMI's (less than 22 kg/m^2^) found the EMG more uncomfortable than the NCS. Possible explanations for the above associations are speculative, but may include pretest expectations associated with health conditions (e.g., older patients are more likely to have polyneuropathy, shown elsewhere in our study to be associated with a greater likelihood of finding the NCS more uncomfortable), or alterations in examination technique dictated by the body habitus of the patient.

Patients referred for evaluation of carpal tunnel syndrome or ulnar neuropathy found the NCS more uncomfortable at a noticeably higher frequency than the baseline study population, and this finding was also apparent in patients whose EDX were consistent with these diagnoses. While this may relate to the difference between the number of nerves and number of muscles tested as described below, it is conceivable that there is a pathological feature of these entities conveying a greater sensitivity to the nociceptive stimulus of an electric shock over that of a needle puncture. It remains unclear why patients whose EDX were normal rated the EMG as more uncomfortable. Patients with diabetes mellitus and hypothyroidism rated the NCS as more uncomfortable at a higher frequency than the baseline study population. This may simply reflect the known association between these disease entities and polyneuropathy, one of the few factors related to a greater likelihood of finding the NCS more uncomfortable than the baseline study population.

Of note, upon retrospective review, not documenting the stimulus intensity could have been a limitation to the study. Those with higher BMI's, carpal tunnel syndrome, ulnar neuropathies, diabetes mellitus, polyneuropathy, or hypothyroidism may have had received higher stimulus intensities thus making them more averse to the NCS. At the same time, we wanted all patients to undergo standard laboratory protocol with a consistent methodology. Controlling factors such as stimulus intensity, needle selection, or particular diagnosis could have skewed our results. Because we did not control for diagnoses, we did have a low sample of patients with motor neuron disease (*n* = 2), and thus the total number of muscles sampled in these patients was less than the total number of nerves sampled. A future study could compare discomfort of EDX testing to the gravity of the diagnosis (i.e., radiculopathies versus motor neuron disease).

As may be expected, a greater number of nerves tested with NCS correlated with a higher frequency of rating the NCS as more uncomfortable, although this relationship did not become apparent until patients had 11 or more nerves tested. Patients in whom the number of nerves tested exceeded the number of muscles tested by four or more also consistently rated the NCS as more uncomfortable. It is notable that patients whose examinations included more muscles tested than nerves clearly rated the NCS as more uncomfortable; this could reflect acclimation to the needle puncture stimulus with increasing exposure. Patients in whom a median-ulnar sensory comparison NCS was carried out rated the NCS as more uncomfortable at a higher frequency than the baseline study population, but as this test is routinely performed for evaluation of carpal tunnel syndrome, this may simply be an epiphenomenon of the dislike of NCS in that disease entity. Patients whose needle examinations included the gastrocnemius or biceps brachii were more likely to rate the EMG as more uncomfortable than the baseline study population, which may simply indicate that these muscles, for a variety of possible reasons, are generally more sensitive to needle puncture stimulus than others.

Patients who are highly anxious about the potential for a painful experience during their EDX may have difficulty fully relaxing and cooperating with the examiner, which may unnecessarily prolong the examination and decrease the utility of the results obtained. Therefore, it would be worthwhile to judiciously utilize any measures that can alleviate pain or anxiety before or during the test. An understanding of which patients are more likely to find the NCS or the EMG more uncomfortable can be useful in this regard. First, in patients for whom the EMG may be predicted to be more uncomfortable (e.g., younger age or lower BMI), previously studied options for pain and anxiety relief should be given greater consideration. These may include premedication with ibuprofen and use of pain reinterpretation techniques (Buckelew et al. 1992). Second, while it is admittedly difficult to carry out a thorough EDX with either more or less nerves or muscles tested than required, it may be beneficial to the patient to make every effort possible to minimize nerves tested or muscles tested in patients predicted to find the NCS or the EMG more uncomfortable, respectively.

## Conclusion

Nerve conduction studies and needle EMG are likely to continue to be important to the practice of clinicians who diagnose and treat neuromuscular disorders. Because of the value of information obtained from these tests, attempts to minimize patient discomfort from the testing are worthy of pursuit. Any information that will allow prediction of discomfort from one test or the other is therefore useful, and our study would indicate that some characteristics of the patients and the test may be utilized in this regard. Our results indicate that older patients, those with higher BMI, and those with polyneuropathy are more likely to find the NCS more uncomfortable. If 11 or more nerves are tested during the examination, these patients will also be more likely to experience greater discomfort with the NCS. Application of this information will allow maximum benefit of this diagnostic tool.
